# Effects of Acupuncture on Explosive Force Production by the Healthy Female Shoulder Joint

**DOI:** 10.1155/2020/8835672

**Published:** 2020-12-01

**Authors:** I-Lin Wang, Yi-Ming Chen, Jun Wang, Rui Hu, Ke-Ke Zhang, Chun-Sheng Ho

**Affiliations:** ^1^The College of Physical Education, Hubei Normal University, Huangshi City 435002, China; ^2^Graduate Institute, Jilin Sport University, Changchun, Jilin 130022, China; ^3^Department of Physical Therapy, College of Medical and Health Science, Asia University, Taichung 41354, Taiwan; ^4^Division of Physical Medicine and Rehabilitation, Lo-Hsu Foundation, Inc., Lotung Poh-Ai Hospital, Yilan 26546, Taiwan

## Abstract

**Background:**

Acupuncture is often used to treat chronic conditions, such as pain. In recent years, given the importance of the explosive forces generated by shoulder muscles for the completion of motor tasks, studies in which nerves were stimulated through acupuncture to increase the explosive forces were conducted. This study explored the effect of acupuncture on explosive force production by the muscles of the female shoulder joint.

**Methods:**

Eighteen healthy women underwent shoulder adduction (Add), abduction (Abd), flexion (Flex), and extension (Ext) tests with an isokinetic measurement system. Acupuncture was used to stimulate the Zhongfu (LU1), Tianfu (LI3), Xiabai (LU4), Binao (LI14), Naohui (SJ13), Jianliao (SJ14), and Xiaoluo (SJ12) points, and electromyography (EMG) signals were recorded before and after acupuncture.

**Results:**

After acupuncture, there was a significant difference in the average maximum work, the average maximum power, the average maximum speed, the total work in Add/Abd and Flex/Ext, the EMG signals, and the stiffness of the muscles in Abd and Ext (*P* < 0.05). There were no significant differences in the average maximum torque in Abd or Flex.

**Conclusion:**

Based on the results, there may be a significant correlation between the manipulation of different acupoints by acupuncture and the average maximum torque and stiffness. Acupuncture may stimulate nerves to activate muscles and induce a postactivation potentiation effect that improves explosive force production. Therefore, acupuncture as an auxiliary tool may increase the explosive forces generated by acupoint-related muscles by stimulating nerves.

## 1. Introduction

Acupuncture was introduced more than 2,000 years ago [1], and it remains an important part of traditional Chinese medicine (TCM). In 2013, acupuncture was used in 183 countries, according to a survey by the World Federation of Acupuncture-Moxibustion Societies (World Health Organization (WHO) report) [2]. Recently, acupuncture has been used to enhance recovery from sports competitions [3]; for example, it has been shown to enhance balance [4] and reduce spasticity [5]. As these examples show, the use of acupuncture is increasingly widespread.

In sports medicine, acupuncture has been preliminarily used to control pain and relieve muscle aches caused by exercise [6], lateral epicondylitis (tennis elbow) [7], knee osteoarthritis [8], low back and neck pain [9], and rotator cuff tendinitis [10]. Previous studies have shown that acupuncture also has positive effects on strength, aerobic training, flexibility, and athletic performance [11]. Overall, the application of acupuncture in sports medicine can improve athletic performance in domains such as muscle strength. Neurochemical, histological, and neurophysiological studies have attempted to elucidate the mechanisms of action of acupuncture [12]. Additionally, many studies in animals and humans have demonstrated that acupuncture can cause multiple biological responses [13]. For example, needling may cause receptors to send neural impulses to the spinal cord or may act on ascending pathways to the brain, causing the release of neurotransmitters that subsequently modulate functions in the brain as well as in the periphery [14]. Therefore, there is a reasonable physiological basis for acupuncture to improve motor function.

Explosive force, the ability to overcome a certain resistance in a short time and with great acceleration, is considered an important indicator of athletic performance [15]. Given the important role of explosive force and movement in athletic performance, many researchers have extensively studied methods related to increased muscle strength and explosive force training methods, which may be useful for improving athletic performance. For example, studies have shown that whole-body vibration (WBV) training can improve strength, power, and jump height [16], and changes in muscle stiffness are believed to be the mechanism through which vibration training improves functional performance [17]. Additionally, there exists a phenomenon of postactivation potentiation (PAP); that is, previous muscle contraction increases subsequent strength and muscle power output beyond the baseline level [18]. Past research on PAP has mainly focused on exploring the effect of the dynamic stimulation of PAP on the performance of the lower extremities, and stimulation can help increase the subsequent explosive force produced by the lower extremities [19]. One of the mechanisms underlying PAP is an increase in the recruitment of higher-order motor units [20]. The same effect may occur when acupuncture stimulates the surface of the skin to induce accelerated extremity reflexes and increased muscle strength [21]. Therefore, acupuncture may induce PAP by stimulating nerves to improve explosive force production.

In a recent study of the effect of acupuncture on the endurance of the female shoulder joint muscles, it was found that acupuncture can increase the excitability of the shoulder joint muscles, delaying muscle fatigue, and increasing muscle endurance [22]. However, the effectiveness in increasing the explosive force of the female shoulder joint has not been confirmed until now. Therefore, this study aimed to explore the effect of acupuncture on explosive force production by the muscles of the female shoulder joint. In this study, we hypothesized that acupuncture can improve the explosive forces generated by the shoulder joint muscles after isokinetic exercise through the corresponding neurophysiological responses.

## 2. Methods

### 2.1. Study Design

The study was registered prospectively at the Chinese Clinical Trial Registry (Registration number: ChiCTR1900025407). Eighteen healthy female subjects (age: 21.2 ± 7.2; weight: 57.6 ± 6.3 kg; height: 164 ± 4 cm) were recruited at JLSU (September 1, 2019, to September 30, 2019). All participants signed informed consent before they participated in the study.

### 2.2. Subjects

Inclusion in this study was restricted to participants meeting the following criteria: age >18 years, the absence of pain in the upper limbs, the absence of a history of muscle disease, and the absence of acupuncture or any medical treatments within the last 6 months. The patients were encouraged not to perform physical exercise for at least 48 hours before the test [6, 23]. The exclusion criteria were upper limb pain, a history of trauma, neuromuscular impairment, uncontrolled epilepsy, epithelial allergy, or any adverse reactions to needles [24]. Participants with a history of significant trauma or systemic inflammatory conditions, such as rheumatoid arthritis, polymyalgia rheumatica, and fracture, were also excluded [25]. The final analysis conducted in this study included eighteen subjects who met the above criteria.

### 2.3. Instruments

An isokinetic training system (Con-Trex MJ; CMV AG, Dübendorf, Switzerland) was used to collect kinetic data on adduction/abduction (Add/Abd) and flexion/extension (Flex/Ext) of the shoulder joint. A portable surface electromyography (EMG) machine with six channels (BTS FreeEMG 300, BTS SpA, Milan, Italy) and disposable circular electrodes with a diameter of 10 mm was used to collect EMG signals (1000 Hz) before and after acupuncture. Disposable stainless-steel needles (0.25 mm × 40 mm, Suzhou Medical Appliance Factory, Suzhou, People's Republic of China) were used for acupuncture.

### 2.4. Acupuncture

**Figure 1:**
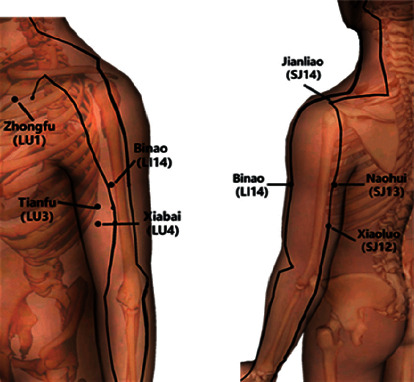
Acupuncture points.

Acupuncture needling was performed by an experienced acupuncturist. Perpendicular needling was carried out bilaterally using sterile disposable needles. The following classical acupuncture points were used in the following order: Zhongfu (LU1), Tianfu (LI3), Xiabai (LU4), Binao (LI14), Naohui (SJ13), Jianliao (SJ14), and Xiaoluo (SJ12) (see Figure 1). The needle was left in place for 20 minutes and then promptly removed. Each needle was rotated at 2 minutes, 5 minutes, and 10 minutes after insertion. The depth of needle insertion depended on the anatomical location of each point and the physical characteristics of each subject (e.g., skin thickness and subcutaneous fat layer thickness) and varied from 5 to 30 mm. De Qi sensation was provoked by manual stimulation (rotation) at the beginning of each session. In TCM, De Qi is a unique sensation of numbness, soreness, heaviness, or tingling that develops at the site of acupuncture, often spreading toward nearby cutaneous areas [21].

### 2.5. Reasons for Acupoint Selection

Studies have shown that the main muscles responsible for shoulder joint Add, Abd, Flex, and Ext are the deltoid anterior (DA), deltoid posterior (DP), and pectoralis (PS), which are all distributed around the shoulder joint [26]. In this study, the Zhongfu (LU1), Tianfu (LU3), Xiabai (LU4), and Binao (LI4) acupoints were located in the anterior Flex and Abd muscle groups of the shoulder joint, while the Jianliao (SJ14), Naohui (SJ13), and Xiaoluo (SJ12) acupoints were located on the Abd and Ext muscle groups of the shoulder joint. Overall, acupuncture points are selected to stimulate nerves and produce PAP according to the principles of TCM.

### 2.6. Protocol

**Figure 2:**
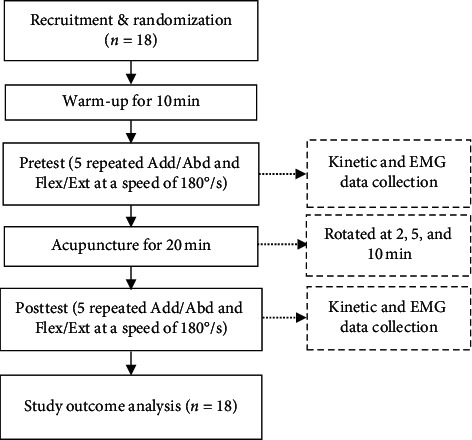
Study flowchart. Note: shoulder joint adduction and abduction (Add/Abd), flexion, and extension (Flex/Ext).

Each subject completed isokinetic strength tests of the shoulder joint muscle group before and after acupuncture, including a pretest and a posttest. We introduced each participant to the entire experimental process to familiarize them with the experimental settings, equipment, and procedures. We simulated the explosive use of muscles of the shoulder joint at a high speed of 180°/s and used isokinetic equipment for standardization. Participants were randomly selected and instructed to warm up for 10 minutes and then rest for 2 minutes. During the break, the skin was cleaned with 75% alcohol. The electrode plates were placed at the approximate center of the belly of each muscle, including the deltoid anterior (DA), deltoid posterior (DP), pectoralis (PS), infraspinatus (ID), and triceps (TC). The electrodes remained attached between trials, so the electrode positions were the same for all trials in each subject. When the electrodes were attached, the participants were asked to relax to avoid the influence of premature muscle activation on the experimental results. The subject took a lateral position: the angle between the seat back and the seat was adjusted to 85°, the rotation angle was adjusted to 15°, the shoulder abduction angle was 90°, the forearm was in a neutral position, the rotation axis of the isokinetic equipment was aligned with the center of the shoulder joint, and the range of motion (ROM) boundary was set. Before testing, the subject first performed three Flex and Ext exercises of the shoulder at an angular speed of 60°/s to become familiar with the movement. Then, the subject performed a group of 15 shoulder Add/Abd and Flex/Ext movements using the isokinetic test system at a speed of 180°/s with verbal encouragement to collect EMG signals as the pretest. After 20 minutes of acupuncture, as with the pretest, EMG signals were collected during another isokinetic test with verbal encouragement. The experimental process is shown in Figure 2.

### 2.7. Data Analysis

The data were further analyzed using MATLAB (version R2016a; MathWorks, Inc., Natick, MA) in terms of the average maximum torque, the average maximum work, the average maximum power, the average maximum speed, the total work and stiffness of the shoulder joint Add/Abd, and Flex/Ext muscle groups and including the EMG signals. Past studies have shown that torque, work, power, and speed can be used as measures of explosive force [27]. Stiffness is another measure of explosive force [28]. Therefore, in this study, we choose these parameters to assess the changes in the explosive forces generated by the shoulder joint muscles. The middle section of the selected EMG signal, lasting 5 s, was recorded as the isometric maximum voluntary contraction (MVC) of each muscle. All EMG signals were processed using specific routines carried out in MATLAB [29]. The EMG signal data are presented as MVC%. The stiffness of the shoulder joint (*K*_joint_) tested was determined by the ratio of the change in the average torque (Δ*T*_joint_) to the change in the angle of the shoulder joint (Δ*θ*_joint_) and was calculated using the following formula:*K*_joint_=(Δ*T*_joint_/Δ*θ*_joint_).

### 2.8. Statistical Analysis

All data were analyzed using SPSS 23.0 software (Chicago, IL, USA). The data are reported as the mean ± standard deviation (SD). The pretest and posttest kinetic data and EMG signals of the shoulder joint muscles were compared using paired *t*-tests. The significance level for all statistical analyses was set at *P* < 0.05.

## 3. Results

**Figure 3:**
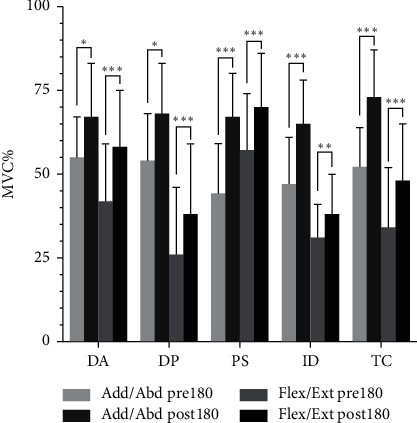
Differences in Add/Abd and Flex/Ext before and after acupuncture. Notes: ^*∗*^*P* < 0.05; ^*∗∗*^*P* < 0.01; ^*∗∗∗*^*P* < 0.001. Shoulder joint adduction and abduction (Add/Abd), flexion, and extension (Flex/Ext). Deltoid anterior (DA); deltoid posterior (DP); pectoralis (PS); infraspinatus (ID); triceps (TC).

All participants successfully completed the study, and the EMG results are presented in Figure 3. There was a significant difference after stimulation in the Add/Abd and Flex/Ext of the DA, Deltoid DP, PS, ID, and TC of shoulder joint (*P* < 0.05) (see Figure 3).

Kinetic data of shoulder joint Add/Abd are shown in Table 1. The average maximum torque of Add significantly increased following acupuncture (+Δ45%, *P* < 0.001). The average work of Add/Abd significantly increased following acupuncture (+Δ40%, +Δ25%, *P* < 0.05). The average power of Add/Abd significantly increased following acupuncture (+Δ74%, +Δ46%, *P* < 0.05). The average maximum speed of Add/Abd significantly increased following acupuncture (+Δ35%, +Δ24%, *P* < 0.001). The total work of Add/Abd significantly increased following acupuncture (+Δ41%, +Δ25%, *P* < 0.05). The total work (Add + Abd) increased following acupuncture (+Δ33%, *P* < 0.001). The stiffness of the joint during Add also significantly increased following acupuncture (*P*=0.02). However, there was no significant difference in the average maximum torque of Abd (*P*=0.076) or the stiffness of the joint during Abd (*P*=0.09).

Kinetic data of shoulder joint Flex/Ext are shown in Table 2. The average maximum torque of Ext significantly increased following acupuncture (+Δ30%, *P* < 0.001). The average work of Flex/Ext significantly increased following acupuncture (+Δ26%, +Δ33%, *P* < 0.001). The average power of Flex/Ext significantly increased following acupuncture (+Δ47%, +Δ52%, *P* < 0.001). The average maximum speed of Flex/Ext significantly increased following acupuncture (+Δ23%, +Δ25%, *P* < 0.001). The total work of Flex/Ext significantly increased following acupuncture (+Δ27%, +Δ35%, *P* < 0.001). The total work (Flex + Ext) increased following acupuncture (+Δ31%, *P* < 0.001). The stiffness of the joint during Ext also significantly increased following acupuncture (*P*=0.002). There was no significant difference in the average maximum torque of Ext (*P*=0.081) or the stiffness of the joint during Flex (*P*=0.112).

## 4. Discussion

### 4.1. Analysis of Kinetic Data before and after Acupuncture

In this study, the average maximum torque of the shoulder joint Add and Ext muscle groups increased after acupuncture. This may indicate that the stimulation of muscle contraction by acupuncture reflects an increase in the ability of the nervous system to drive the muscle to produce maximum torque. Acupuncture stimulates nerves to recruit more motor units and/or stimulate currently active motor units at higher frequencies, and segmental reflex rings may also be involved [30]. Therefore, in this study, acupuncture stimulation of the nerves may have accelerated limb reflexes and recruited additional motor units to increase muscle contraction and increase the average maximum torque. However, the average maximum torque of the shoulder joint Flex and Abd muscle groups was not effectively improved after acupuncture. This may be related to the specificity of acupuncture points. A previous study has shown that acupuncture stimulation of muscles can induce changes in motor cortex excitability and that the level of motor cortex excitability is related to the selected muscle and the needle insertion point [21]. Additionally, stimulating different acupuncture points will trigger different activation modes in the brain [31]. Therefore, in this study, the average maximum torque of the shoulder joint Flex and Abd muscle groups could not be effectively improved, possibly because of acupoint specificity.

The average maximum power, average maximum work, average maximum speed, and total work of the shoulder joint Add/Abd and Flex/Ext muscle groups increased after acupuncture. This is likely because acupuncture can effectively increase muscle function, such as power, work output, and contraction speed. Previous studies have suggested that the stimulation of nerves by acupuncture can change the excitability of cortical motor neurons [32] as well as lead to the recruitment of more motor units and/or the generation of higher-frequency activity of already active motor units [30]. The effect was similar to that of PAP. A study has shown that PAP can increase work and power output [18]. One mechanism for this phenomenon is the phosphorylation of myosin regulatory light chains. Additionally, myosin light chain kinase is responsible for the formation of the actin-myosin complex to provide more adenosine triphosphate (ATP) [20], which increases the muscle's ability to do work. Another study used PAP principles to explain why short-term exercise improves performance. The reason is that the stimulation of motor nerves can enhance the tolerance of muscles to aerobic exercise, delay muscle fatigue, and improve exercise performance [33]. Another possible mechanism is that the PAP phenomenon causes an increase in the recruitment of motor units, increasing the efficiency and degree of neurotransmitter transfer and recruiting higher-level motor units (type II muscle fibers), thereby increasing the ability of muscles to produce force and increasing subsequent explosive performance [34]. When acupuncture is applied to specific points on the body surface for stimulation, it can activate multiple pathways in the nervous system, accelerate the feedback speed of neurons, and stimulate muscles to respond quickly [35]. Therefore, in this study, acupuncture of the acupoints of the shoulder joint Add/Abd and Flex/Ext muscle groups improved the ability of the muscles to perform work and increased the explosive force of the shoulder joint, which may be related to the benefits of PAP.

### 4.2. Analysis of Surface EMG Signals before and after Acupuncture

EMG signals were significantly enhanced after acupuncture. Research suggests that manual acupuncture (MA) or electroacupuncture (EA) at particular acupoints activates afferent fibers that send signals to the spinal cord [1]. The regulation of motor neuron activity is realized through the input of the spinal cord and the upper spinal cord. Short-term and long-term acupuncture stimulation of somatosensory afferent nerves can induce neuromuscular excitability changes at the level of the spinal cord, especially the upper spinal cord [36]. Additionally, acupuncture stimulates peripheral *α* motor neurons by stimulating peripheral nerves to generate nerve impulses, increasing the transmission rate of spinal nerve impulses and the firing frequency of motor units [20, 34]. In another study, the excitability of spinal motor neurons increased significantly after repeated acupuncture with H reflex measurement in the musculus soleus [37]. Therefore, in this study, acupuncture may have increased nerve impulses and activated acupuncture-related muscles by stimulating nerves, eventually enhancing the EMG signal at the acupuncture site.

### 4.3. Effect of Joint Motion on Muscle Stiffness

In this study, the stiffness of the shoulder Add and Ext muscle groups significantly increased after acupuncture. Past studies have shown that, among the factors that affect joint stiffness, the most important is the degree of muscle activation [38]. Additionally, soft tissues, such as tendons, also increase muscle stiffness as they contract or stretch [39]. Therefore, in this study, acupuncture stimulated nerves to activate related muscles and increased the average maximum torque and stiffness of the shoulder joint Add and Ext muscle groups.

### 4.4. Limitations

The limitations of this study are that the effects of fake acupuncture were not assessed, and muscle blood flow was not measured to determine the mechanism of acupuncture.

## 5. Conclusion

In this study, isokinetic testing and acupuncture were used to investigate the effects of acupuncture on the explosive muscle force of the female shoulder joint during Add/Abd and Flex/Ext. The results show that acupuncture may induce PAP and increase the average work, power, torque, and speed of the muscles related to the acupoints, thereby increasing the explosive forces generated by the shoulder joint muscle groups. The results support the hypothesis that acupuncture can improve the explosive forces generated by the shoulder joint muscles after isokinetic exercise through corresponding neurophysiological responses. Therefore, acupuncture may be used as an alternative medicine to improve physical performance in sports medicine. However, the average maximum torque of the shoulder Flex and Abd groups did not show a statistically significant difference after acupuncture. This may be related to the selected acupoints, so the specificity of acupuncture and potentially related factors need to be further explored in future studies.

## Figures and Tables

**Table 1:** Mean (SD) shoulder joint Add/Abd before and after acupuncture.

Add/Abd	Pre 180	Post 180	*P*
	Mean ± SD	Mean ± SD	
Average max torque, Add (Nm/kg)	0.42 ± 0.12	0.61 ± 0.13	<0.001
Average max torque, Abd (Nm/kg)	0.34 ± 0.13	0.33 ± 0.25	0.76
Average work, Add (J/kg)	0.50 ± 0.19	0.70 ± 0.15	<0.001
Average work, Abd (J/kg)	0.44 ± 0.17	0.55 ± 0.15	0.001
Average power, Add (W/kg)	0.34 ± 0.15	0.59 ± 0.13	<0.001
Average power, Abd (W/kg)	0.28 ± 0.10	0.41 ± 0.11	0.001
Average max speed, Add (deg/s ∗ kg)	1.98 ± 0.52	2.68 ± 0.35	<0.001
Average max speed, Abd (deg/s ∗ kg)	1.73 ± 0.38	2.15 ± 0.27	0.002
Total work, Add (J)	426.76 ± 151.15	602.52 ± 105.61	<0.001
Total work, Abd (J)	376.23 ± 127.02	468.94 ± 122.31	0.002
Total work, Add + Abd (J)	802.99 ± 274.49	1071.46 ± 209.04	<0.001
Stiffness, Add	0.003 ± 0.007	0.936 ± 1.267	0.02
Stiffness, Abd	0.002 ± 0.001	0.049 ± 0.090	0.09

Differences were considered significant when *P* < 0.05. Add/Abd indicates shoulder joint adduction/abduction.

**Table 2:** Mean (SD) shoulder joint Flex/Ext before and after acupuncture.

Flex/Ext	Pre 180	Post 180	*P*
	Mean ± SD	Mean ± SD	
Average max torque, Flex (Nm/kg)	0.3 ± 0.11	0.36 ± 0.19	0.081
Average max torque, Ext (Nm/kg)	0.47 ± 0.12	0.61 ± 0.07	<0.001
Average work, Flex (J/kg)	0.43 ± 0.09	0.54 ± 0.10	<0.001
Average work, Ext (J/kg)	0.55 ± 0.16	0.73 ± 0.12	<0.001
Average power, Flex (W/kg)	0.30 ± 0.09	0.44 ± 0.10	<0.001
Average power, Ext (W/kg)	0.46 ± 0.18	0.70 ± 0.12	<0.001
Average max speed, Flex (deg/s ∗ kg)	1.77 ± 0.32	2.18 ± 0.31	0.002
Average max speed, Ext (deg/s ∗ kg)	2.25 ± 0.56	2.81 ± 0.32	<0.001
Total work, Flex (J)	364.91 ± 79.07	462.34 ± 93.19	<0.001
Total work, Ext (J)	467.99 ± 127.00	632.92 ± 118.68	<0.001
Total work, Flex + Ext (J)	832.90 ± 194.67	1095.26 ± 203.42	<0.001
Stiffness, Flex	0.002 ± 0.002	0.154 ± 0.304	0.112
Stiffness, Ext	0.004 ± 0.005	0.112 ± 0.096	0.002

Differences were considered significant when *P* < 0.05. Flex/Ext indicates shoulder joint flexion/extension.

## Data Availability

The datasets used and analyzed in the current study are included in this article.
